# Improved pH-Responsive Release of Phenformin from Low-Defect Graphene Compared to Graphene Oxide

**DOI:** 10.1021/acsomega.1c03283

**Published:** 2021-09-14

**Authors:** Abdelnour Alhourani, Jan-Lukas Førde, Lutz Andreas Eichacker, Lars Herfindal, Hanne Røland Hagland

**Affiliations:** † Department of Chemistry, Biosciences and Environmental Technology, University of Stavanger, 4021 Stavanger, Norway; ‡ Centre for Pharmacy, Department of Clinical Science, University of Bergen, 5007 Bergen, Norway; § Department of Internal Medicine, Haukeland University Hospital, 5021 Bergen, Norway

## Abstract

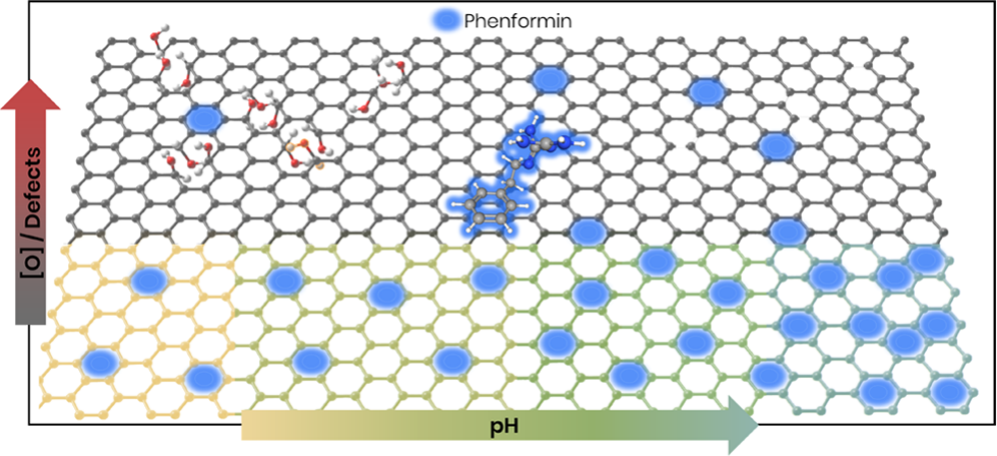

Graphene-based drug carriers provide a promising addition to current cancer drug delivery options. Increased accessibility of high-quality graphene made by plasma-enhanced chemical vapor deposition (PE-CVD) makes it an attractive material to revisit in comparison to the widely studied graphene oxide (GO) in drug delivery. Here, we show the potential of repurposing the metabolic drug phenformin for cancer treatment in terms of stability, binding, and pH-responsive release. Using covalent attachment of poly(ethylene glycol) (PEG) onto pristine (PE-CVD) graphene, we show that PEG stabilized graphene nanosheets (PGNS) are stable in aqueous solutions and exhibit higher binding affinity toward phenformin than GO. Moreover, we experimentally demonstrate an improved drug release from PGNS than GO at pH levels lower than physiological conditions, yet comparable to that found in tumor microenvironments.

## Introduction

Graphene, a two-dimensional (2D) hexagonal carbon isolated from graphite crystals,^1,2^ has been investigated as a possible carrier of drugs and genomic materials for enhanced therapeutic effect.^1,3,4^ Using graphene as a drug carrier is possible because of the large surface area of graphene sheets containing pi electrons,^1^ which allows for high adsorption of hydrophobic and aromatic drugs by noncovalent interactions, such as pi–pi interactions.^3,5^

Graphene oxide (GO) has been particularly tested for cancer drug delivery,^3,6−8^ due to its stability in aqueous solutions^9^ caused by basal plane defects resulting in a high number of oxygen-rich groups on its surface.^10^ GO is often produced via liquid-phase exfoliation of graphite, usually based on Hummer’s method.^11,12^ However, its high acidity and abundance of reactive functional groups, such as epoxides and carboxylic acids,^2,13,14^ lead to low compatibility with physiological buffers,^3,15^ as well as cytotoxicity by increasing intracellular levels of reactive oxygen species (ROS).^16−18^ On the other hand, graphene produced by chemical vapor deposition (CVD) renders fewer sheet defects and, therefore, lower oxygen content,^9,12^ especially under high temperatures^10^ or plasma-enhanced (PE-CVD) production.^19^ The lower number of defects, however, increases the hydrophobicity of graphene and thereby lowers the dispersibility in aqueous solutions.^20^

One of the main purposes of dedicated drug delivery systems is to allow for controlled drug delivery and release at a target tissue or organ. This minimizes unwanted side effects and increases exposure of drug at the tissue of interest, which makes the therapy more efficient compared to conventional drug-based therapy.^4,21,22^ Graphene possesses preferred properties over the clinically used liposomes, as its carbon-based structure is impermeable to other molecules, reducing the risk of cargo leakage while requiring less demanding storage conditions.^23−28^ However, its inability to form a stable dispersion in aqueous solutions, especially for nonoxidized graphene, has hampered its use compared to other drug delivery platforms.

Besides size limitations due to intravenous administration, delivery of nanosized carriers requires that graphene sheets are small enough to pass the leaky vessel endothelium in tumors to accumulate utilizing the enhanced permeability and retention (EPR) effect.^21,29,30^ While the production of graphene usually renders polydisperse sheet sizes, multiple protocols to obtain a narrow size separation of graphene down to a nanometer range have been described previously,^31−36^ making graphene an ideal candidate to fully exploit the EPR potential. Furthermore, the lower pH found in tumor microenvironments as a result of rapid cancer cell growth^37^ can be used for targeted drug release. GO has previously shown amphiphilic properties stemming from hydrophobic basal regions among the hydrophilic functional groups,^38,39^ allowing for targeted release of loaded drugs at lower pH.^6,40,41^ However, in contrast to GO, this ability remains to be investigated for CVD graphene, with its lower defect levels and fewer functional groups, as the pH-dependent release from GO has been attributed to the lower binding with these groups^8,42^ affected by the protonation of the loaded drug in lower pH.^41,43,44^

To test graphene’s potential as a drug carrier, we used phenformin, a biguanide antidiabetic drug,^45,46^ that has previously been tested in cancer models using micelles as a drug carrier.^45^ The analogue metformin, commonly used to treat diabetes type 2, has recently been studied extensively for its potential as a cancer drug, inhibiting key metabolic pathways needed for cell growth.^47^ Phenformin acts similar to metformin in cancer cell lines, but has higher potency.^43,46−48^ Phenformin was discontinued in diabetes treatment in the early 1980s due to undesired side effects,^48^ but its newly discovered beneficial effects in cancer treatment may outweigh the previously experienced risks.^46,49−53^ Importantly, these side effects could be further mitigated by the use of a dedicated drug carrier, such as graphene nanosheets. On a structural level, phenformin contains a guanidine group, which could form hydrogen bonds between the amine groups in guanidine and the carboxyl groups on graphene sheets.^54,55^ In addition, phenformin contains a phenol residue that could bind to graphene through pi–pi interactions due to delocalized electrons on the graphene surface.^54,55^ Drug delivery of phenformin’s analogue, metformin using carbon nanotubes in cancer cells has been reported,^56^ as well as controlled metformin drug release using GO hydrogels in mice.^57,58^ Moreover, GO has recently been used to selectively deliver metformin to triple-negative breast cancer,^59^ demonstrating increasing interest and relevance for improving biguanide drug delivery using graphene-based drug carries.

Here, we report on the stability and binding properties of two graphene-based drug carriers, PEGylated graphene nanosheets (PGNS) and GO, in relation to the biguanide drug phenformin. This work is highly relevant for expanding the cancer drug repertoire and holds promise for overcoming challenges related to using metabolic drugs in cancer treatment.

## Results and Discussion

### Characterization of PGNS and GO

To increase the solubility of PE-CVD graphene, we covalently attached poly(ethylene glycol) (PEGylation) onto PE-CVD graphene sheets. The PEGylation approach was used to retain the defect-free graphene properties of PE-CVD, while obtaining an irreversible increase in water solubility, but without introducing oxidations of the basal plane as in GO. This should preserve the pi electrons that are necessary for drug adsorption and yielding lower oxygenation levels in the PEGylated graphene nanosheets (PGNS). Throughout this work, we compare the PGNS to a commercially available GO from ACS materials.

The atomic ratio of oxygen to carbon, determined by X-ray fluorescence (XRF), was around 5 times lower in PGNS compared to GO (Table 1). This suggests that the PE-CVD production method, followed by the PEGylation process, does not introduce a high number of oxygen-carrying groups compared to that found in GO.

**Table 1:** Comparative Characterization of PGNS and GO

		PGNS	GO
Oxygen content (XRF carbon int. weighted)		0.023 ± 0.04	0.125 ± 0.08
*D*_τ_ in diH_2_O (μ^2^/s)	pH 7.5	1.326 ± 0.18	1.131 ± 0.2
	pH 6.5	0.996 ± 0.2	1.114 ± 0.24
	pH 5	0.417 ± 0.03	0.718 ± 0.09
ZP in diH_2_O (mV)	pH 7.5	–21 ± 4	–37.6 ± 6
	pH 6.5	–15 ± 2	–36.13 ± 6
	pH 5	– 5 ± 2.5	–28.3 ± 2.9
AFM graphene layer height (nm)		5–15 nm	1–3 nm (detection limit)

a XRF characterization to estimate the relative oxygen ratio (*N* = 3, ±SD) normalized to carbon intensity-weighted content, dynamic light scattering (DLS) to assess diffusion coefficient (*D*_τ_) and ζ-potential (ZP) variation against pH change (*N* = 3, ±SD), and atomic force microscopy (AFM) to determine sheet height. Abbreviations: graphene oxide (GO), PEGylated graphene nanosheets (PGNS), X-ray fluorescence (XRF), dynamic light scattering (DLS).

Dynamic light scattering (DLS) was used to compare the effect of pH on the diffusion coefficient (*D*_τ_) and ζ-potential (ZP) of GO and PGNS. The ZP and *D*_τ_ measurements showed no significant variation at pH 7.5 and 6.5 between PGNS and GO (Table 1 and Figure S1). However, at pH 5, the *D*_τ_ was decreased for GO and was 3-fold lower for PGNS compared to pH 7.5. This substantial change in *D*_τ_ indicates changes in either the shape or the hydrodynamic radius of the particle. The ZPs of PGNS and GO at physiological pH were −21.5 and −37.5 mV, respectively. The more negative ZP of GO is probably linked to its higher oxygen content compared to PGNS. Furthermore, acidic pH had a minimal effect on the ZP of GO compared to PGNS, the latter being almost neutral at pH 5 (Figure S1c). When dispersed in fetal bovine serum over 5 days, GO showed more signs of protein adsorption than PGNS. Thereafter, both GO and PGNS maintained their *D*_τ_ and ZP levels compared to day 1 (Table S1).

**Figure 1:**
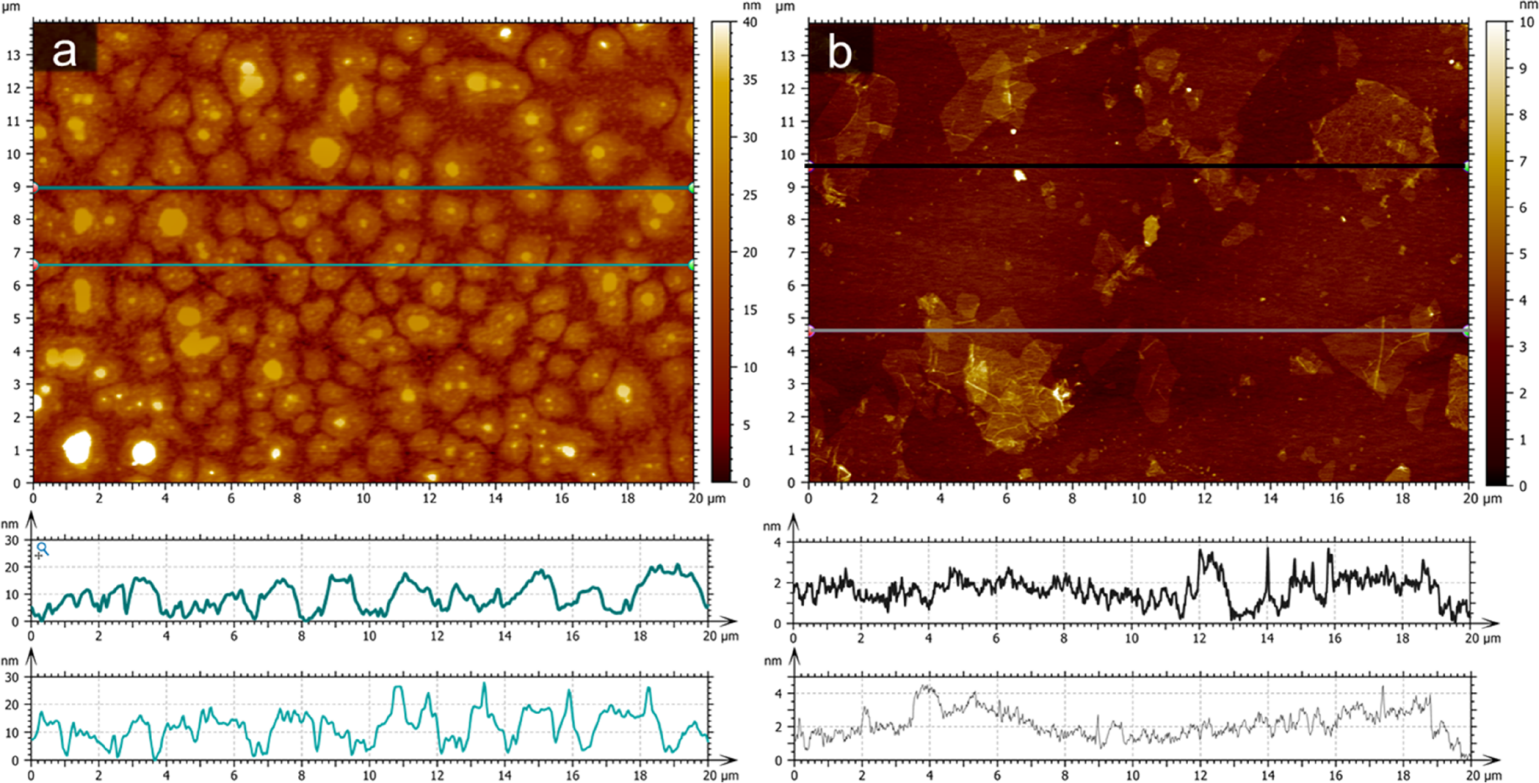
AFM characterization of (a) PGNS and (b) GO showing individual sheets of single and few layers with height using pseudo-coloring. Abbreviations: graphene oxide (GO), PEGylated graphene nanosheets (PGNS), and atomic force microscopy (AFM).

Atomic force microscopy (AFM) was used for the shape characterization of both materials. The PGNS sample was found to consist of sheets that are relatively similar in size (mean diameter = 262 ± 75 nm) but, on average, showed smaller diameters than GO (mean diameter = 448 ± 226 nm) (Figures 1a and S2). The AFM scan also revealed possible solvent residues on top of the PEGylated graphene sheets, which could be caused by the solvent being trapped by the PEG chains during evaporation. The remaining solvent complicates the estimation of the layer numbers. GO was found to consist of primarily monolayer sheets of vastly varying size compared to PGNS (Figures 1b and S2). There was a high dispersity between sheet sizes, ranging from few nanometers to multiple micrometers in the largest sheet diameter.

The PE-CVD graphene used in this study was vertically grown on the substrate and therefore rendered few (∼less than 10) layers thick sheets that were initially hydrophobic and not dispersible in water. However, after PEGylation, the resulting graphene sheets were stable and dispersed in deionized water for more than 1 week and could easily be redispersed by gentle shaking (Figure S3).

### PGNS Provides Better Aqueous Dispersion Stability Than GO When Loaded with Phenformin

**Figure 2:**
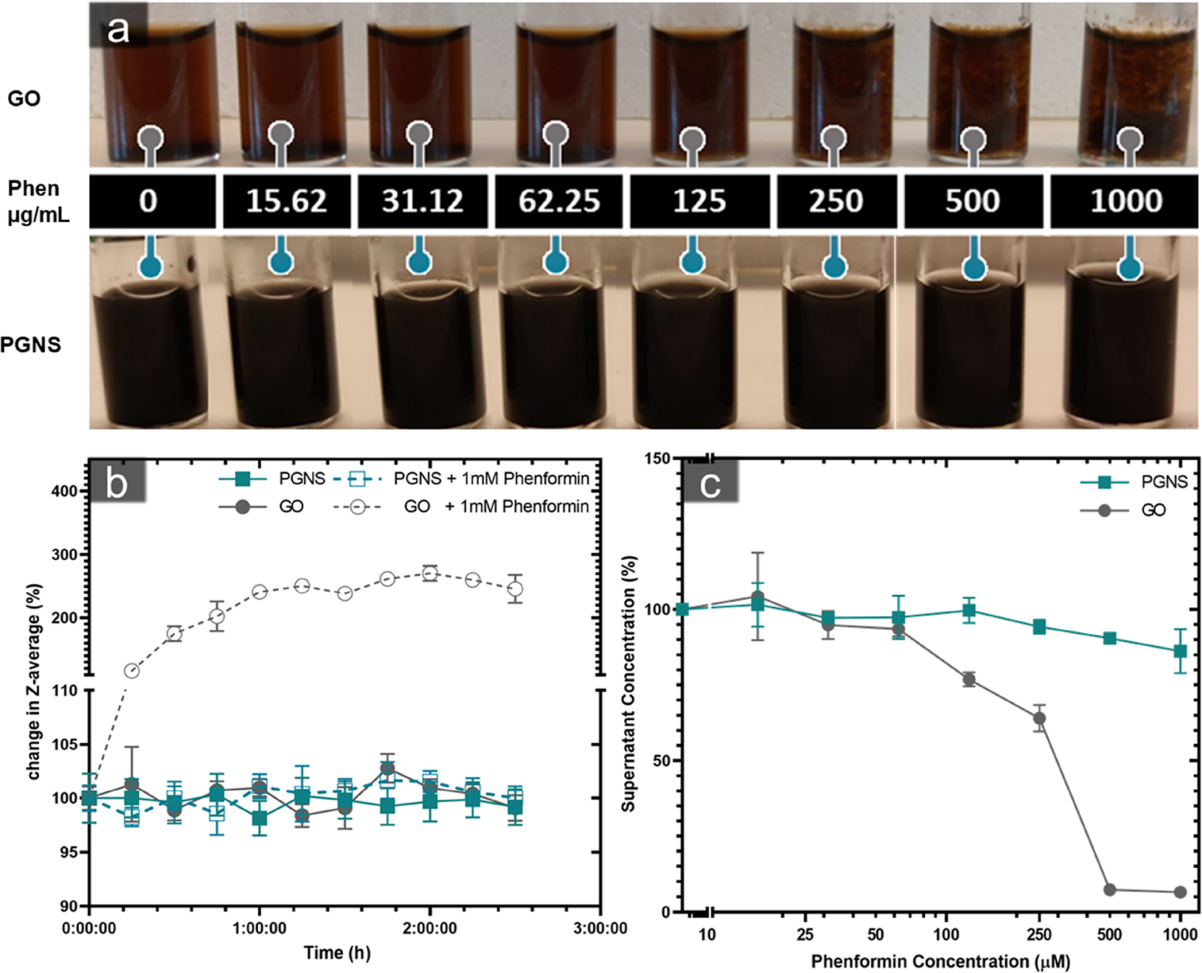
Reduced stability of GO compared to PGNS after the addition of phenformin to water dispersion. (a) Images of glass tubes containing GO (top) and PGNS (bottom) with a serial increase in phenformin concentrations (left: 0–1000 μg/mL) showing visible agglomeration in GO after 24 h. (b) Time-resolved intensity-weighed *Z*-average size estimation of GO and PGNS with and without the addition of phenformin, represented as a change from *T* = 0 (*N* = 3, ±SD). (c) GO and PGNS supernatant concentrations 24 h after the addition of increasing concentrations of phenformin. The supernatants were collected after gentle centrifugation of samples, and the integrated area under the absorbance spectrum between 400 and 900 nm is displayed as % from control to indicate concentration (*N* = 3, ±SD). Abbreviations: graphene oxide (GO), PEGylated graphene nanosheets (PGNS).

An important aspect of evaluating the use of nanoparticles for drug carrier application is its colloidal stability, particularly after drug loading. To assess the stability, time-resolved DLS measurements were conducted for GO and PGNS with the addition of phenformin. The underlying stability of both dispersions, prior to the addition of phenformin, is represented by the consistent baseline of intensity-based size measurements over a period of 165 min (Figure 2b). It can therefore be concluded that GO and PGNS maintained a stable size distribution in solution over the recorded measurement, with no detected graphene agglomerates formation. However, upon the addition of 1 mM phenformin to both dispersions, the *Z*-average size measurements of GO increased during the first hour, indicating the formation of larger agglomerates in the solution. These effects were not seen in PGNS within the same time frame under the same conditions.

To find the threshold of GO agglomeration induced by phenformin and identify possible agglomeration in PGNS that went undetected by DLS, we added increasing concentrations of phenformin (15.6 μM up to 1 mM) to both dispersions. Visually detected destabilization of GO sheets and formation of agglomerates were seen at concentrations of 250–1000 μM phenformin (Figure 2a) and confirmed by microscopy (Figure S4). Such agglomeration was not observed in PGNS at the same phenformin concentrations. A titration experiment was conducted to measure the extent of GO and PGNS stability upon increasing phenformin concentrations. Here, a centrifugation step was introduced to isolate the dispersed supernatant of GO and PGNS in a solution mixed with increasing concentrations of phenformin (15.6–1000 μM) after 24 h of interaction time. The graphene concentrations in the collected supernatants were calculated by integrating the area under the absorbance spectrum in the visible range. A decrease in measured supernatant graphene concentration would signify agglomeration due to the faster sedimentation of larger formed particles under equal centrifugal forces. We found that PGNS had lower levels of agglomeration compared with GO at concentrations of up to 1 mM phenformin (Figure 2c). Agglomeration of GO was dose-dependent and was detected with phenformin concentrations down to 100 μM. PGNS also showed concentration-dependent agglomeration but at a slower rate than GO (Figure 2c). The addition of 250, 500, and 1000 μM phenformin resulted in supernatant concentrations relative to controls of 94, 90, and 86% for PGNS versus 64, 7.3, and 6.4% for GO, respectively (Figure 2c).

Depending on the pH of the solution, phenformin exists in the solution as either a divalent or monovalent ion. Therefore, addition of phenformin will increase the ionic strength of the solvent, in this case, water, and is expected to reduce the repulsive colloidal stability of graphene by reducing the strength of the electrical double layer surrounding the sheets. This double layer is also expected to be larger in GO due to higher oxygen content leading to increased electron density, which in turn would be more effective in overcoming the attractive forces that would otherwise bring the graphene sheets together.^60^ For example, the higher stability in GO compared with reduced GO is due to decreased strength of the electrical double layer in reduced GO.^60^ Similarly, the lower oxygen content on PE-CVD graphene would also result in a decreased electric double layer, making it unstable in water without the conjugation of PEG. However, contrary to GO, the addition of phenformin showed negligible effect on PGNS stability, most likely due to the PEGylation.

In support of this, other groups have demonstrated the agglomeration effect seen in GO by increasing the monovalent Na^+^ ion concentrations to a critical coagulation concentration of 44–60 mM.^60,61^ Furthermore, reports of divalent ions Ca^2+^ and Mg^2+^ interaction with GO^61^ show critical coagulation concentrations comparable to the 1 mM phenformin concentrations used in our study, which supports our findings that phenformin compromises GO stability in solution. Moreover, it has been shown previously that steric stabilization using PEG prevents ion-mediated agglomeration,^62^ supporting our observations that phenformin induced less agglomeration in PGNS than GO. The significance of this effect in the bloodstream is critical for the choice of nanoparticles in drug delivery. The ionic destabilization, here represented by phenformin, gives an indication of the stability of graphene if exposed to the naturally occurring electrolytes in the bloodstream. Blood electrolytes, such as sodium and calcium cations, maintained at concentrations over 100 and 2 mmol/L respectively,^63^ may pose a concern toward the stability of repulsively stabilized nanoparticles such as GO. However, from our data (Figure 2), this can be overcome using steric stabilization.

### Kinetics of Phenformin Adsorption onto PGNS and GO

Phenformin has the potential to bind with a graphene sheet by either pi interactions with the hydrophobic basal plane of graphene or via the interaction between the amine group of the phenformin and the carboxylic groups of graphene. Microscale thermophoresis (MST), time-correlated fluorescence imaging, and time-resolved absorption measurements were used to understand the kinetics and affinity of the interaction of phenformin with graphene dispersions.

**Figure 3:**
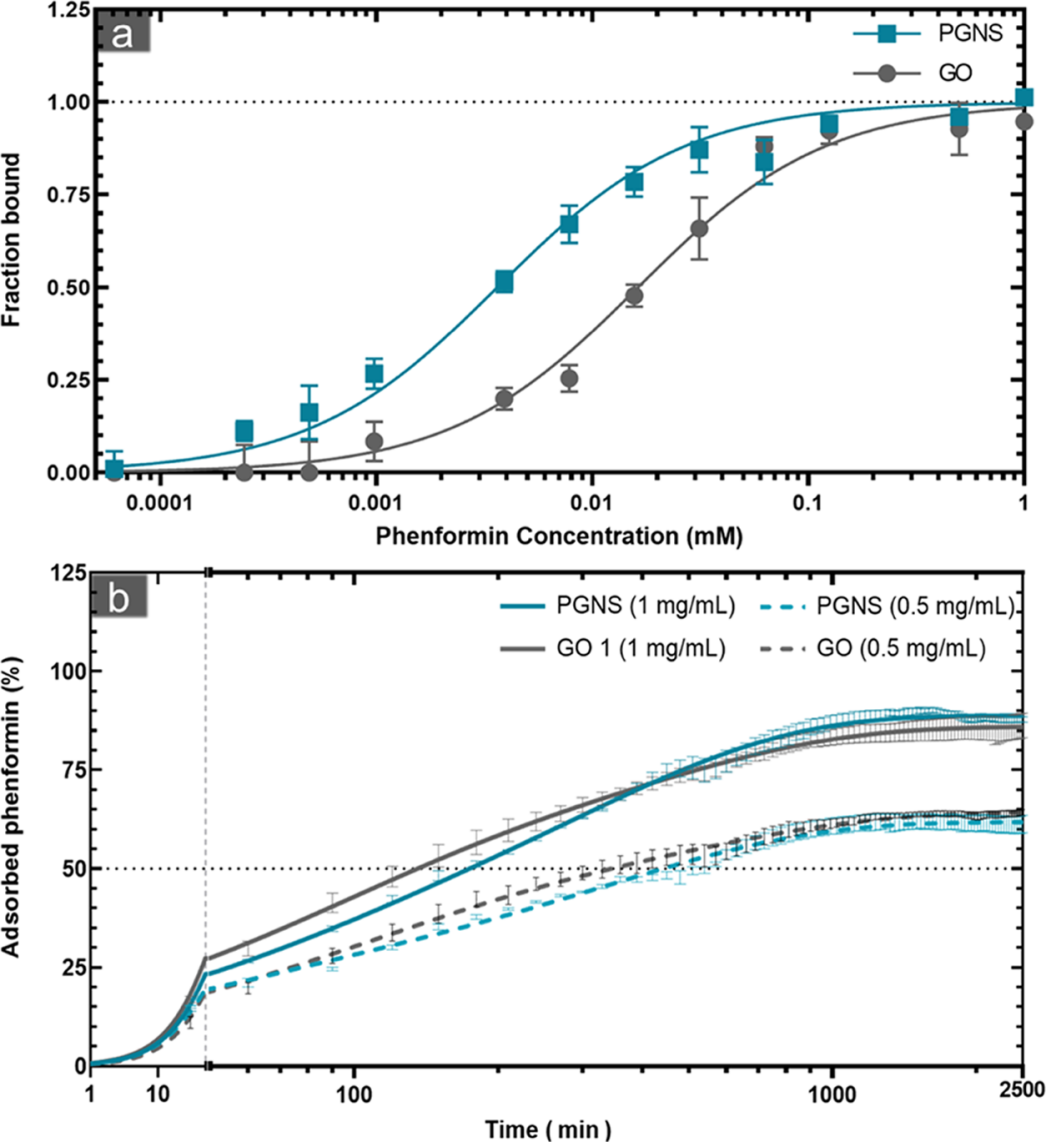
Binding affinity and kinetics of phenformin from PGNS and GO. (a) MST-derived fractional binding of a series concentrations of phenformin up to 1 mM onto GO and PGNS, specific binding curves with Hill’s slope. (b) Kinetic measurements reflecting the % of phenformin adsorption on GO and PGNS. The analysis was done using GO and PGNS at two different concentrations of 0.5 and 1 mg/mL. The graphs show the continued measurements of free phenformin removed over 24 h from an initial (*T* = 0) concentration of 50 μM (10.26 μg/mL). (*N* = 2 and 3, ±standard error of mean (SEM) for PGNS and GO, respectively). Abbreviations: microscale thermophoresis (MST), graphene oxide (GO), PEGylated graphene nanosheets (PGNS).

MST traces were collected after 24 h of phenformin interaction in a titration series (0–1 mM) with PGNS and GO to calculate the binding at each concentration. As MST relies on the temperature-induced changes in fluorescence to calculate binding, PGNS was covalently labeled with an Atto-488 dye, while the autofluorescence of GO was sufficient to calculate the binding. Hill’s slope-fitted fractional binding curves showed 4.5 times higher binding affinity of phenformin toward PGNS compared to GO with *k*_d_ values of 3.625 ±0.551 and 16.25 ± 2.138 μM, respectively (Figure 3).

To monitor the binding kinetics at an equilibrium state, the adsorption of 50 μM phenformin was monitored over time on 0.5 and 1 mg/mL of GO and PGNS, respectively. There was rapid drug adsorption within the first 4–8 h (Figure 3), where GO dispersions reached 50% binding 1.3 times faster than PGNS at both concentrations tested (Table 2). However, PGNS reached equilibrium before GO, 1.4 times faster at 1 mg/mL, and 2 times faster at 0.5 mg/mL. This indicates a rapid binding mechanism of phenformin to GO that becomes saturated as phenformin adsorption reaches ∼50%, a mechanism apparently less predominant in PGNS. Such binding could reflect the direct interaction between the phenformin amines and the carboxyl groups. Therefore, a two-phase association model was used to calculate the contribution of this fast adsorption phase for GO and PGNS. Approximately 25% of the total phenformin binding was attributed to the fast phase in PGNS compared to 50% in GO. This relative increase could also be explained by the amine carboxyl interaction.

**Table 2:** Phenformin Kinetic Diffusion Parameters for PGNS and GO

	PGNS		GO	
Concentration (mg/mL)	1	0.5	1	0.5
Time to plateau (h)	18.5	20.5	26	41
Time to 50% (h)	2.86	7.23	2.23	5.62
Fast diffusion ratio (%)	24.55%	29.51%	51.65%	54.25%

a Recorded times needed for each condition to reach an adsorption milestone; two-phase association kinetics fit results after 48 h phenformin adsorption measurements to calculate the contribution of each phase of the adsorption. Abbreviations: graphene oxide (GO), PEGylated graphene nanosheets (PGNS).

**Figure 4:**
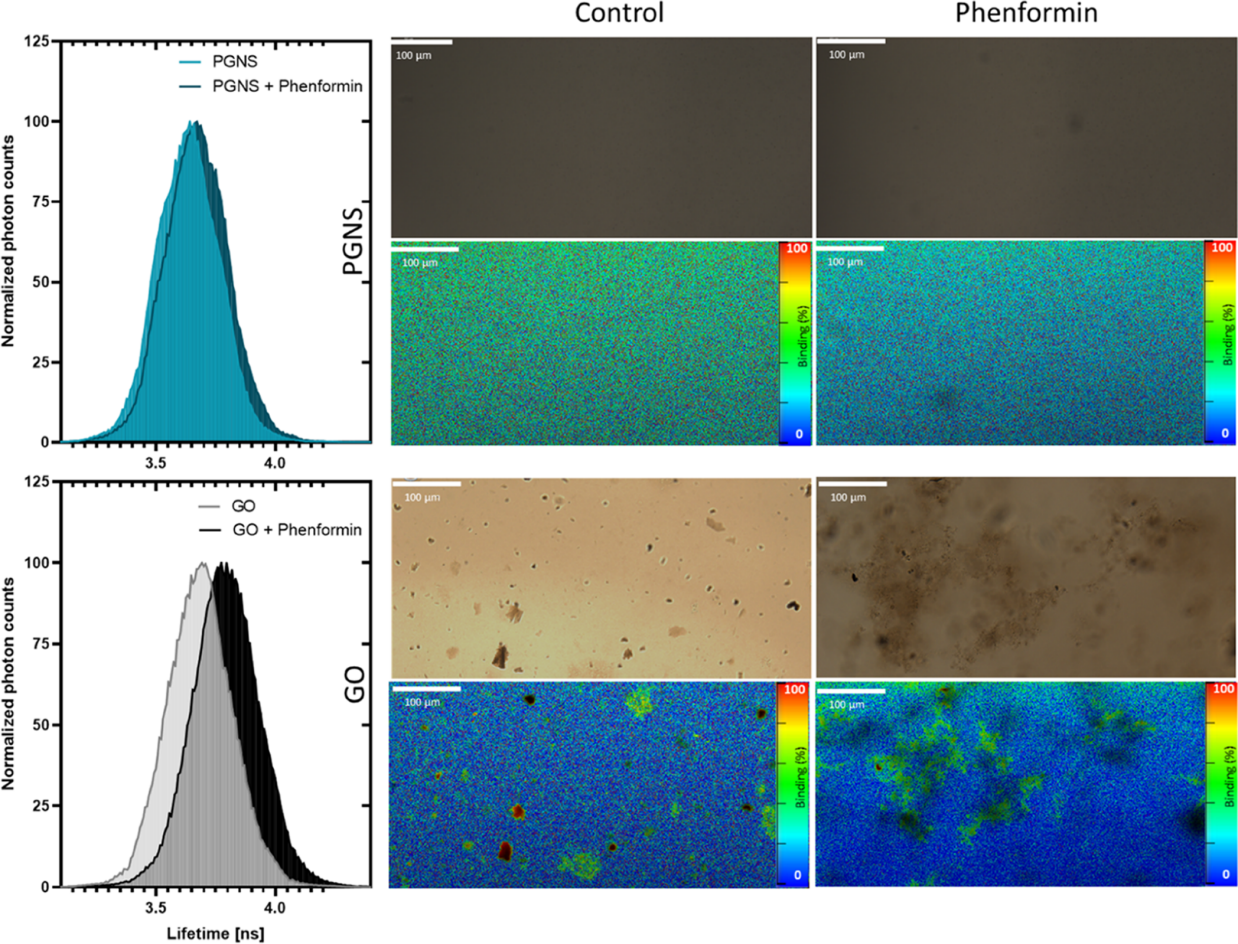
Fluorescence lifetime distributions of fluorescein mixed with PGNS and GO before and after adding 1 mM phenformin (left). Representative images at 20× magnification using bright-field microscopy and specially resolved FLIM-FRET with pseudo-color coding of fluorescein binding efficiency using GO and PGNS as photon acceptors before and after the addition of 1 mM phenformin (right). Abbreviations: microscale thermophoresis (MST), graphene oxide (GO), PEGylated graphene nanosheets (PGNS), fluorescence lifetime imaging microscopy-fluorescence resonance energy transfer (FLIM-FRET).

The binding was studied further as a function of the fluorescence lifetime of fluorescein. Graphene is known to quench the fluorescence of fluorescein donor molecules allowing their use as a fluorescence lifetime imaging microscopy–fluorescence resonance energy transfer (FLIM-FRET) pair.^64,65^ The initial binding of equal fluorescein concentrations onto PGNS and GO was 32.15 and 17.66%, respectively. Since the lifetime of fluorescein is affected by pH (Figure S5), we tracked the change of fluorescein binding onto PGNS and GO after the addition of phenformin. The binding was reduced to 20.3 and 6.37% in PGNS and GO, respectively. A spatial resolution of the binding efficiency showed that the displacement of fluorescein by phenformin appears to be more considerable in the nonagglomerated regions in GO, suggesting a higher phenformin binding in these regions (Figure 4).

In addition to agglomeration, the difference in oxygen content and the extent of surface defects could play a critical role in the discrepancy of phenformin interactions with GO versus PGNS. The measured 5-fold higher oxygen content in GO (Table 1) means more sheet defects and consequently disturbed pi electrons.^10^ Thus, the probability of carboxylic groups, and their interaction with phenformin’s amines, should increase. This interaction is detectable by DLS in the absence of graphene (Figure S6). On the other hand, the removal of oxygen correlates with an increase in the sp^2^ hybrid orbital fraction on the graphene surface.^66^ Therefore, a less defected basal plane as in PGNS would allow for more pi–pi and cation–pi interactions with phenformin. However, the lower number of carboxylic groups on PE-CVD graphene limits their interaction with external amines compared to GO. In addition, a proportion of these groups are converted into amides during the covalent attachment of PEG in PGNS, decreasing their concentration even further. In contrast, the addition of the oxygen-rich PEG arms in PGNS adds a new mechanism and binding opportunity for phenformin. This may happen as we found that the ZP of PEG alone is attenuated after phenformin addition (Figure S7).

### Dissociation Rates of Phenformin from Graphene at Different pH Levels

The effect of pH on the release profile of phenformin from GO and PGNS was studied after 24 h of interaction. The amount of unbound phenformin was measured at pH values that simulate shifts between normal tissues and tumor microenvironment of pH 5, 6.8, and 7.4 with increasing graphene concentrations.

**Figure 5:**
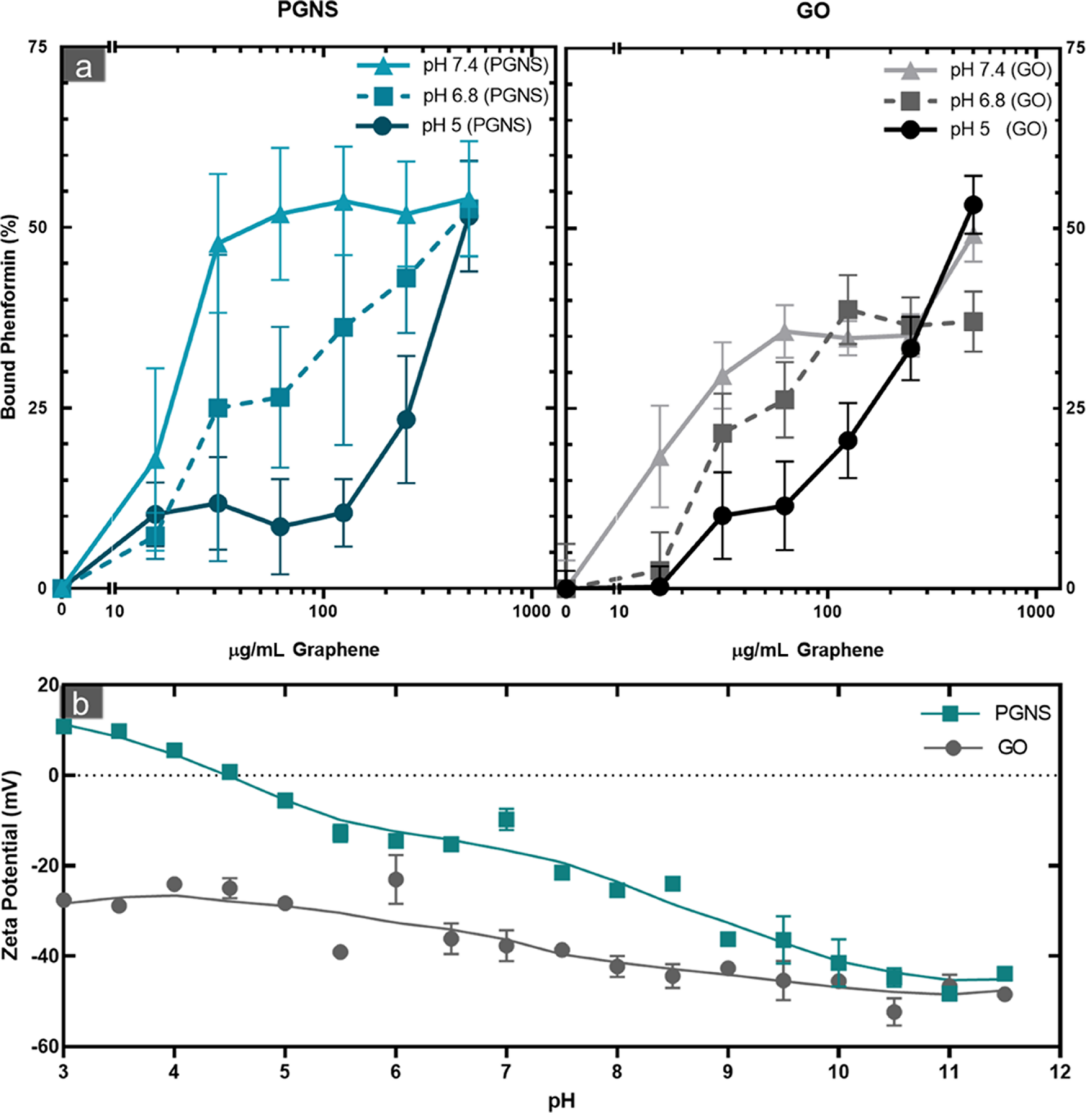
pH-dependent release of phenformin from PGNS and GO. (a) Percentage (%) of bound of phenformin at pH 5, 6.8, and 7.4 is represented at increasing concentrations of PGNS and GO after 24 h measured by high-performance liquid chromatography (HPLC) determination of the unbound filtrate (±SEM, *N* = 3). (b) ZP values of GO and PGNS water dispersions as a function of pH (±SEM, *N* = 3). Abbreviations: high-performance liquid chromatography (HPLC), ζ-potential (ZP), graphene oxide (GO), PEGylated graphene nanosheets (PGNS).

A change in phenformin adsorption capacity onto graphene was affected by pH in both PGNS and GO (Figure 5a). There was an increased release of phenformin in acidic pH compared to physiological pH 7.4. This increase was on average 2.2 and 4.4 times higher at pH 5 than pH 7.4 in GO and PGNS, respectively. More specifically, the release of phenformin from GO was increased 24.2% (±7.1%) and 14.2% (±5.7%) at pH 5 compared with 7.4 at concentrations of 62.125 and 125 μg/mL, respectively. In comparison, the increase in PGNS was 35.2% (±5.9%) and 35.4% (±5.2%) for the same concentrations.

These results correspond to the change in ZP of PGNS and GO at pH values ranging from 3 to 11.5 (Figures 5b and S1). The shift toward positive ZP corresponding to more basic pH levels was steeper in PGNS compared to GO, spanning over a 3.8-fold increased charge range in the tested pH values. An increased positive electrostatic potential at the shear planes of PNGS could cause an enhanced release of the positively charged phenformin at acidic pH values, compared to GO. The decrease in negative charges appeared to correlate with the pH-dependent release of the adsorbed phenformin in PGNS and GO (Figures 5b and S1). Thus, as the pH drops lower than physiological level, similar to that observed in the microenvironment of tumor or lysosomes,^21^ more phenformin is released.

The difference in the modular affinity between phenformin and GO or PGNS at acidic versus basic pH values could be due to a combination of different interaction mechanisms between phenformin and graphene. In GO, the degree of protonation of carboxylic groups on GO and guanidinium in phenformin might be a key factor. At neutral pH levels, carboxylic groups are more likely to interact with the protonated biguanide core of phenformin.^67^ However, this type of interaction is less likely under acidic conditions. Carboxyl groups are increasingly protonated, leading to lower binding and the consequent release of phenformin. Previous studies demonstrating the pH-dependent drug release of the cancer drug doxorubicin from GO have ascribed this effect to weakened hydrogen bonding under acidic conditions.^41,43,44,68,69^ While it could be argued that PGNS is also affected by the same mechanism, the lower density of carboxylic groups on its surface should limit this contribution compared to GO. Therefore, this might not explain the enhanced pH-dependent release observed in our experiment using PGNS. However, the higher capacity to participate in pi interactions due to the low-defected surface, such as in PGNS, makes it more relevant for the adsorption of phenformin than in GO. The interaction with the basal plane of graphene could occur through either pi–pi interactions with the phenol ring or cation–pi interactions with the amine group of phenformin. Cation–pi interactions, in particular, are known to increase in strength with increasing pH.^70^ Thus, the increased probability of establishing pi–interaction can provide a reason for the higher pH-responsiveness in PGNS compared to GO.

Additionally, the PEG arms in PGNS could be involved in the pH-responsive binding of phenformin. While PEG could interact with phenformin itself (Figure S7), this ability can be limited by possible PEG adsorption onto the graphene surface. Poly(ethylene glycols) have been shown to adsorb onto activated carbons in a pH-dependent manner, where the adsorption was at its lowest at pH 5.^71^

## Conclusions

We show that using covalent PEGylation, PE-CVD graphene sheets can be modified to overcome its hydrophobicity and provide better colloidal stability in the presence of the metabolic drug phenformin. Furthermore, we show that phenformin adsorption capacity is increased in the PEG-functionalized graphene compared to GO, likely due to higher pi- and PEG-mediated interaction possibilities. Most importantly, the pH-responsive phenformin release is not only conserved in nonoxidized graphene sheets but appears to be enhanced in PGNS. Finally, this work shows that PEGylated pristine graphene may be a better carrier than oxidized graphene for drug delivery of phenformin and warrants further exploration in cancer model systems.

## Methods

### Preparation of PGNS and GO

PE-CVD graphene flakes (10 mg, as obtained from CealTech, Stavanger, Norway) were dispersed in DiH_2_O and sonicated in a glass vial for 10 min until dispersion. The pH of the dispersion was adjusted to 5.5 with HCl. Graphene (1:5 wt %) and monofunctional 2K mPEG-Amine (50 mg, Biochempeg, MA) were added to the washed PE-CVD graphene and sonicated with the graphene for 5 min. The cross-linking was then mediated by adding 1.5 mg of 1-ethyl-3-(3-dimethylaminopropyl) carbodiimide (EDC) (Merck KGaA, Darmstadt, Germany) to the mixture under sonication for 30 min. The dispersion was left under constant agitation overnight. The excess EDC and mPEG were then removed by dialysis for 48 h using 30 mL of Slide-A-Lyzer Dialysis Cassettes (Thermo Scientific, Massachusetts) in DiH_2_O that is replaced after 2, 6, and 24 h. The PEG–graphene suspension was sequentially sonicated in a bath sonicator and then washed with DiH_2_O using 100 kDa Vivaspin (Sartorius AG, Göttingen, Germany) centrifugal concentrators three times. Thereafter, the UV–vis spectrum of the filtrate was controlled for the absence of absorbance peaks differentiating from a blank DiH_2_O control (WL: 200–900 nm).

Commercially available graphene oxide prepared by modified Hummer’s method (GNO1W001, ACS Material, LLC, Pasadena) was used in this study. A 1 mg/mL water dilution was made in DiH_2_O that is then cross-filtrated using Vivaflow 50 (Sartorius AG, Göttingen, Germany) to neutralize the pH and eliminate any existing contaminants.

### Characterization

#### X-ray Fluorescence (XRF)

PGNS and GO suspension (50 μL) was placed dropwise on a silver membrane filter (Cat. No. 1145348, Osmonics, Inc., Minnetonka). The samples were dried at 50 °C for >30 min and measured on an S4 PIONEER X-ray spectrometer (Bruker AXS GmbH, Karlsruhe, Germany) the following day. To calculate the relative oxygen content, the intensity values from carbon and oxygen of blank filters were subtracted from the samples. To compensate for variations in the amount of PGNS and GO placed on the filters, the oxygen content of all filters was normalized based on the measured carbon content. The relative oxygen content was then calculated by dividing the resulting relative oxygen content of GO by PGNS.

#### Atomic Force Microscopy (AFM)

For analysis, 20 μL of the suspensions were placed on a mica substrate and evaporated at 50 °C for >30 min and allowed to cool before analysis. The sample was analyzed in repulsive mode on an MFP-3D-BIO (Asylum Research, Oxford Instruments, California). Noise filtration was performed using two-dimensional fast Fourier transform (2D-FFT) filtering in Gwyddion 2.57. Analysis of sheet diameter distribution is based on the radius from the center of mass calculated from a minimum of 150 sheets segmented by height.

#### Dynamic Light Scattering (DLS)

DLS-based surface charge and intensity-weighted sizes of PGNS and GO were measured using Zetasizer Nano ZSP (Malvern Panalytical, Malvern, United Kingdom) with an inline MPT-2 degassed titrator. The titration compartment containing 10 mL of GO or PGNS was kept under constant agitation and real-time pH measurement to adjust the pH at each titration step using HCL and NaOH.

### Stability Study

#### UV–Vis Spectroscopy

Phenformin (Cayman Chemical, Michigan, United States) was added in increasing concentrations (15 μM to 1 mM) to 1 mg/mL of PGNS and GO suspensions. After 24 h, 3000 RCF centrifugation for 10 min was performed to sediment larger agglomerates. The supernatants were collected, and their concentration was measured using UV–vis spectroscopy by integrating the area under the spectrum in the visible range between 400 and 900 nm.

#### DLS

Size measurement of PE-CVD graphene and graphene oxide at 100 μg/mL concentration was carried out continuously over time using DLS (Malvern Panalytical Ltd, UK) for 150 min with and without 1 mM phenformin. The calculated sizes were normalized to the initial measured size at *T* = 0.

### Phenformin Binding Kinetics

#### UV–Vis Kinetic Measurements

In plastic UV cuvettes, 1 mL of 50 μM phenformin (the concentration based on a pilot experiment showing equilibrium reached within 24 h under same parameters without graphene) was separated from 400 mL of water suspensions of PGNS and GO at concentrations of 500 and 1000 μg/mL using a 20K MWCO RC membrane. Continuous absorbance measurements at 233 nm were taken (15 min intervals) using a Shimadzu UV-1800 UV–visible spectrophotometer. The decrease in absorbance correlated with the decrease of phenformin in the lower compartment and its binding to graphene. The adsorption of phenformin is then calculated after background subtraction (based on water-only internal control) and the ratios of interpolated measured values by the maximum phenformin concentration of 10.26 μg/mL.

#### Fluorescence Lifetime-Based FLIM-FRET

Lifetimes of (15 μg/mL) fluorescein (Merck, Darmstadt, Germany) in exposure to 100 μg/mL of graphene with and without the addition 0.5 and 1 mM of phenformin was recorded using a Leica TCS SP8 falcon platform (Leica Microsystems, Mannheim, Germany). The lifetime decays were collected and fitted to a two-exponential tail fit decay to calculate the intensity-based mean lifetimes and the FLIM-FRET changes corresponding to phenformin doses (Tables S2 and S3).

#### Microscale Thermophoresis (MST)

First, fluorescent PGNS were produced similar to the production of PGNS described in the Preparation of PGNS and GO section. However, 10% of the added Monofunctional 2K mPEG-Amine was substituted with an ATTO-488-Amine (ATTO-TEC, Siegen, Germany) dye to obtain a green fluorescent PGNS for MST analysis. The *k*_d_ of GO and PGNS was determined via microscale thermophoresis (MST; Monolith NT.115, Nano Temper). Series concentrations of phenformin (0–1 mM) in DiH_2_O were mixed with GO or Atto-488-PGNS to a final concentration of 50 μg/mL. Afterward, each of the mixtures was pulled into a capillary and set into the Monolith NT.115 capillary compartment. MST power (40%) was used in combination with the blue LED power to determine the MST traces. The change in the thermophoresis of the fluorescence correlates with higher phenformin binding at each concentration used. MST traces derived fraction bound values were plotted against the phenformin molar concentration in MO. Affinity analysis software was used and *k*_d_ was calculated from the dose–response curve.

#### High-Performance Liquid Chromatography (HPLC)

The binding of phenformin at different pH levels is determined via HPLC. Phenformin (50 μM) was mixed with a series dilution of PGNS and GO (15–500 μg/mL) in triplicate at 3 pH levels of 5, 6.8, and 7.4. After 24 h, 300 μL of the mixture was moved to a 10 kDa MWCO filtration plate and centrifuged over a collection plate at 500 rpm until complete filtration. Each filtrate (100 μL) was then measured for unbound phenformin at 233 nm using HPLC (Hitachi High Technologies, Tokyo, Japan) over Phenyl-Hexyl 2.7 μm column using a gradient of DiH_2_O and acetonitrile (30:70%) as the mobile phase.

## Supporting Information Available

ζ-Potential pH dependence for graphene nanoparticles (Figure S1); AFM-based size estimation of graphene nanoparticles (Figure S2); PGNS and GO prolonged dispersion stability in water (Figure S3); bright-field microscopy images at 5× magnification of 100 μg/mL of GO and PGNS at concentrations of 0, 0.5, and 1 mM of phenformin (Figure S4); fluorescence arrival time histograms showing the effect of pH on the lifetime of fluorescein in 2-(*N*-morpholino)ethanesulfonic acid (MES) buffer (Figure S5); ζ-potential changes toward more positive charges of carboxymethylcellulose (Figure S6); ζ-potential of mPEG-amine in water before and after adding phenformin (Figure S7); stability of GO and PGNS in DI water versus fetal bovine serum (FBS) (Table S1); lifetime fit values for fluorescein decays with GO and PGNS (Table S2); FLIM-FRET fit values for fluorescein decays with GO and PGNS (Table S3) (PDF)
