# Supplementary material for: Improved pH-Responsive Release of Phenformin from Low-Defect Graphene Compared to Graphene Oxide
Source: ACS Omega. 2021 Sep 14;6(38):24619–29. doi: 10.1021/acsomega.1c03283 (PMC8482513; doi:10.1021/acsomega.1c03283)
Supplement: Supplementary file 1 — ao1c03283_si_001.pdf [file ao1c03283_si_001.pdf]

Improved pH-responsive release of phenformin from low-defect graphene compared to graphene oxide

Abdelnour Alhourani[1], **Jan-Lukas Førde**[2,3], **Lutz Andreas Eichacker**[1], **Lars Herfindal**[2], **Hanne Røland Hagland***[1]

[1]*Department of Chemistry, Biosciences and Environmental Technology*, University *of Stavanger, Stavanger, Norway*

[2]*Centre for Pharmacy, Department of Clinical Science, University of Bergen, Bergen, Norway*

[3]*Department of Internal Medicine, Haukeland University Hospital, Bergen, Norway*

Supporting information

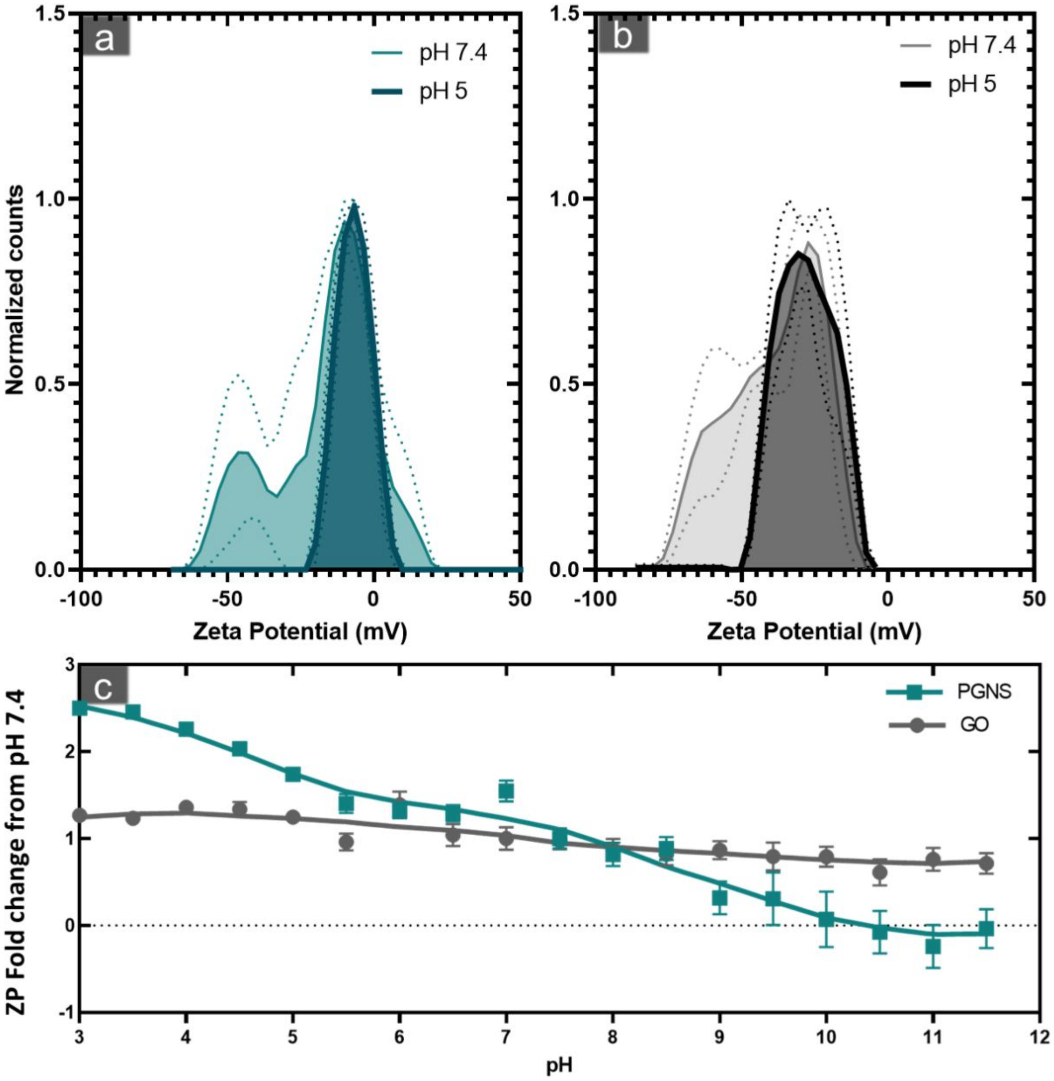

Figure S1 *Zeta potential pH dependence for graphene nanoparticles. Normalized distributions for (a) PGNS and (b) GO. (c) pH-dependent ZP curves of GO and PGNS water dispersions represented as fold changes from ZP value at pH 7.4*

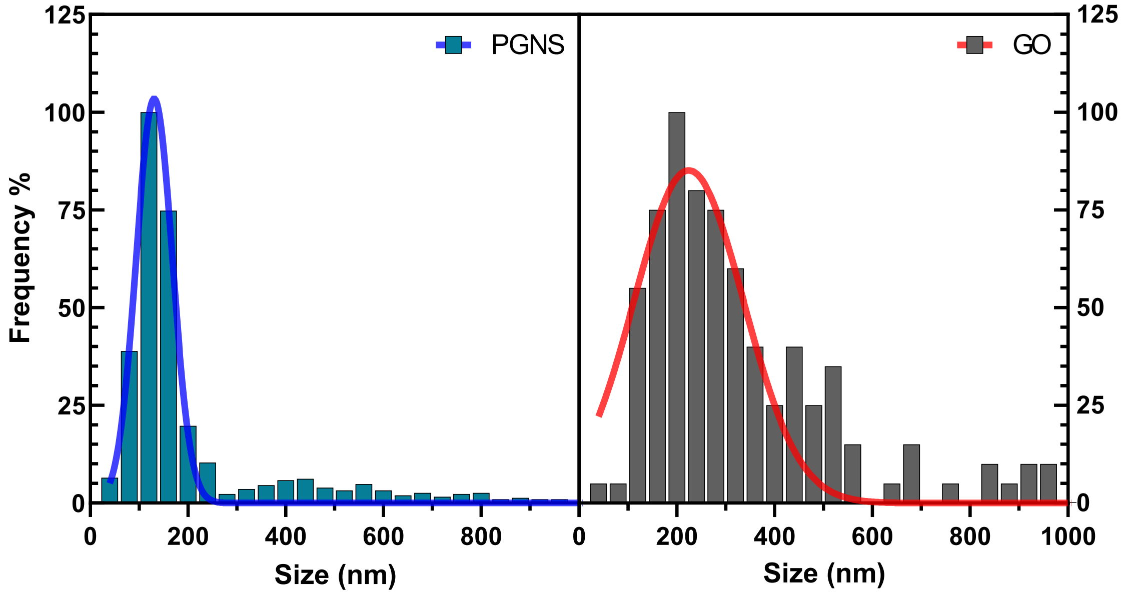

Figure S2 *Afm-based size estimation of graphene nanoparticles. Size distribution for PGNS (Cyan) and GO (grey) measured by atomic force microscopy with overlayed Gaussian distribution fits as blue and red lines, respectively.*

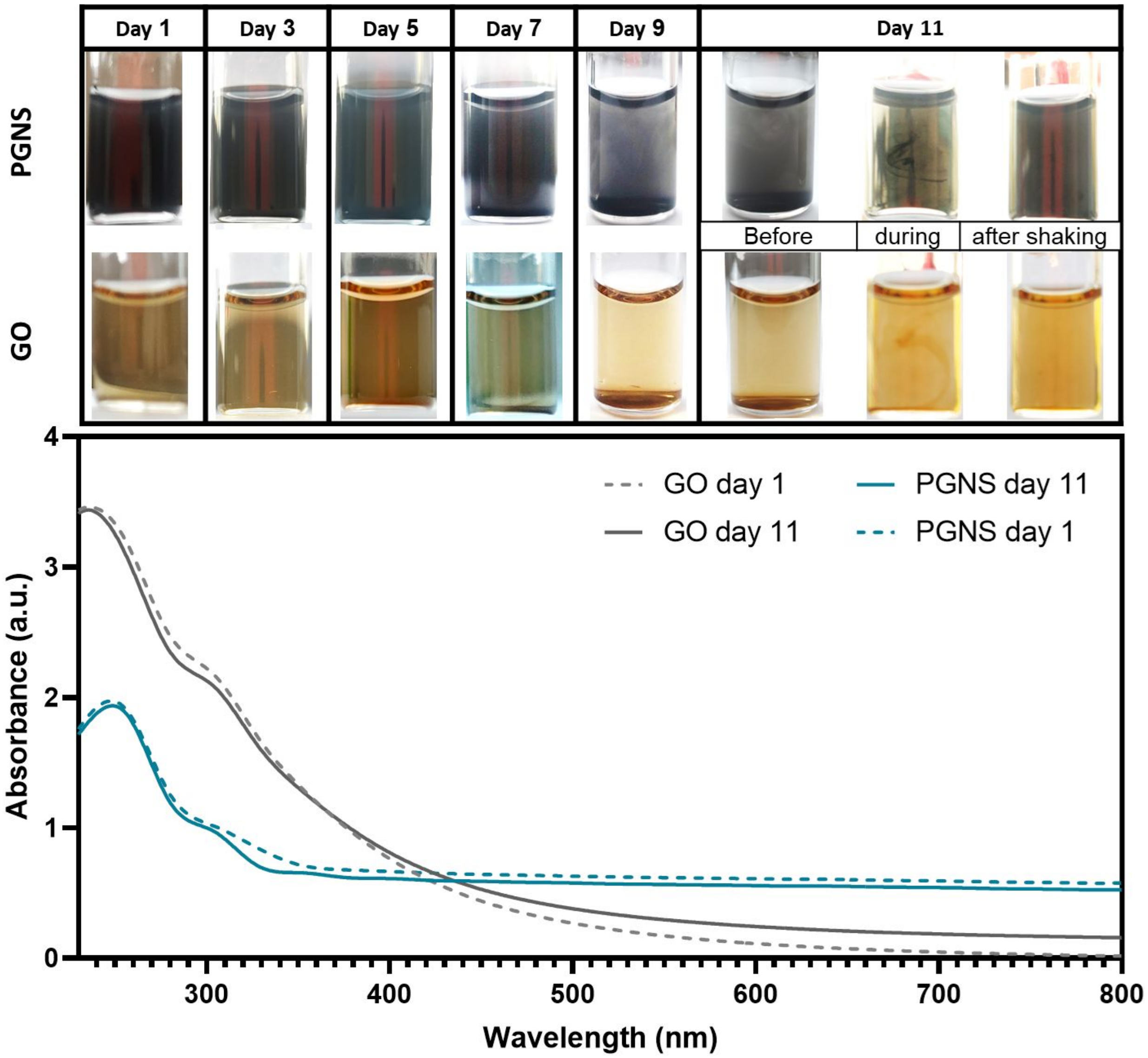

Figure S3 *PGNS and GO prolonged dispersion stability in water. Photographic evidence demonstrating the stability of PGNS compared to GO over 11 days in addition to the corresponding spectra for parallel samples at day 1 and 11 left in the measurement cuvettes without any shaking.*

Table S1 *Stability of GO and PGNS in Di-water versus fetal bovine serum (FBS)*

		PGNS	***GO***
Dτ (μ²/s)	**diH2O**	1.326 ± 0.18	1.131 ± 0.2
	**FBS day 1**	1.22 ± 0.09	1.96 ± 0.1
pH 7.5	**FBS day 5**	1.26 0.01	1.98 0.13
ZP (mV)	**diH2O**	-21± 4	-37.6 ± 6
	**FBS day 1**	- 19 ± 1.5	-7.19 ±0.4
pH 7.5	**FBS day 5**	-20.23 ±1.29	-9.21± 0.5

Diffusion coefficient (Dτ) and zeta potential (ZP) changes in diH2O and FBS for GO and PGNS(N=3, ±SD). PGNS maintains similar Dτ and ZP, while possible protein adsorption onto GO increases its Dτ and attenuates its ZP. After five days in FBS, no significant changes were observed in either PGNS or GO

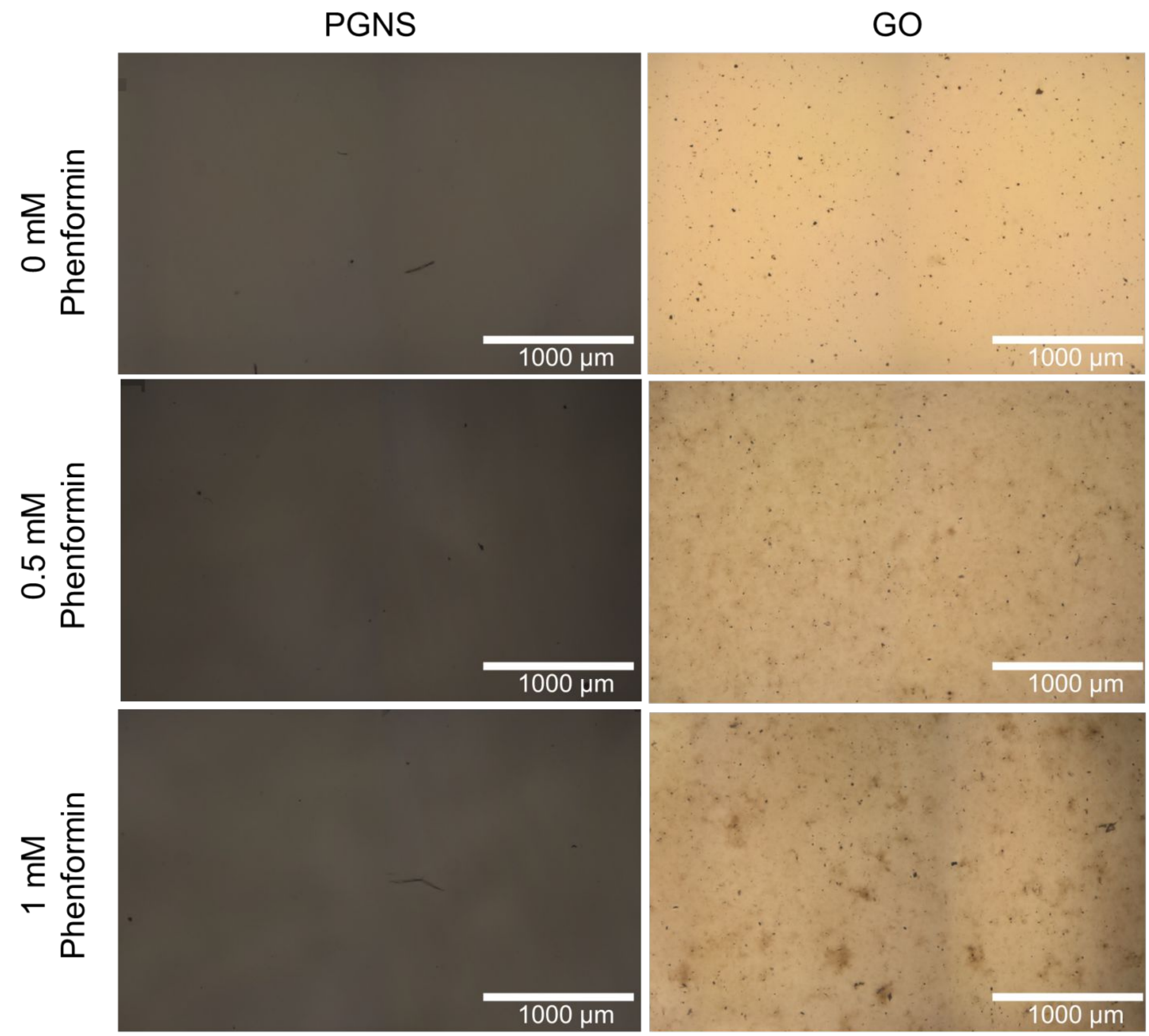

Figure S4 *Bright field microscopy images at 5x magnification of 100 µg/ml of GO and PGNS at concentrations of 0, 0.5, and 1 mM of phenformin.*

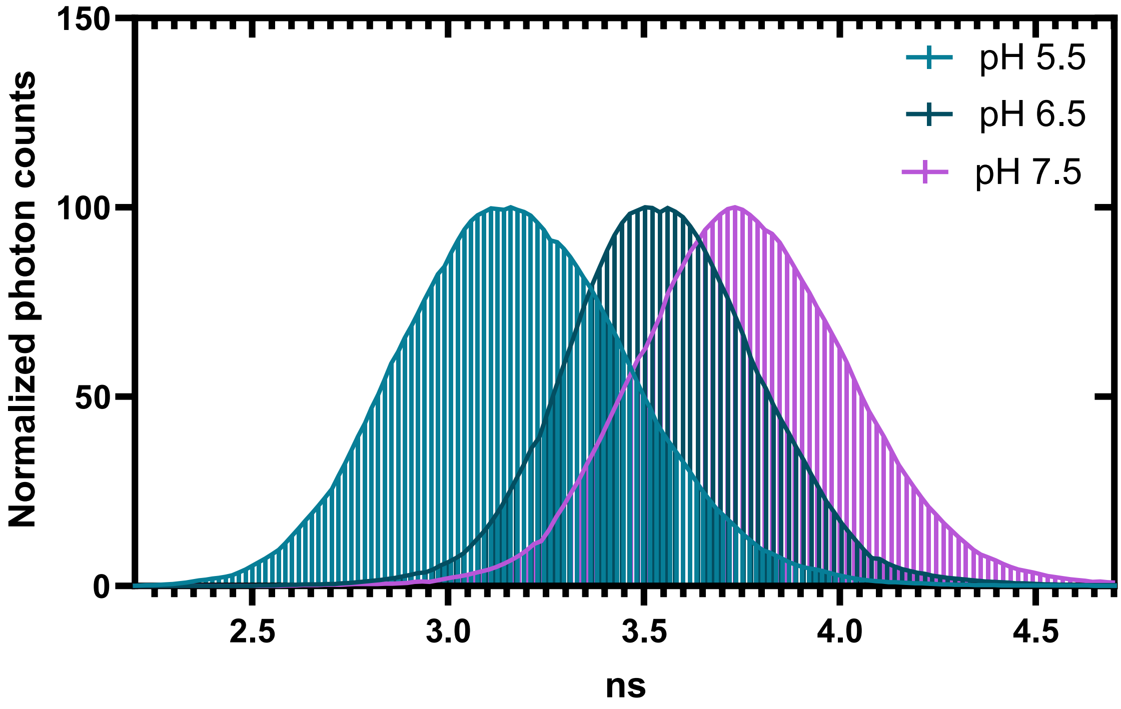

Figure S5 *Fluorescence arrival time histograms showing the effect of pH on the lifetime of fluorescein in MES buffer.*

Table S2 *Lifetime fit values for fluoresceine decays with GO and PGNS.*

	PGNS			GO		
**phenformin (mM)**	0	0.5	1	0	0.5	1
**τ_1 (ns)**	3.143	2.992	2.656	2.990	1.761	0.901
**τ_1 SD (ns)**	0.057	0.056	0.073	0.107	0.154	0.156
**Δ τ_1 (ns)**	0.000	-0.152	-0.487	0.000	-1.228	-2.089
**Δ τ_1 SD (ns)**	0.057	0.056	0.073	0.107	0.154	0.156
**τ_{avg} (ns)**	3.764	3.750	3.801	3.858	3.923	3.936
**τ_{avg} sd (ns)**	0.003	0.004	0.005	0.003	0.007	0.053
**Δ τ_{avg} (ps)**	0.000	-14.273	36.852	0.000	65.407	77.700
**Decay χ^2**	0.873	1.149	0.959	1.049	0.897	1.900
**Binding %**	32.151	30.396	20.301	17.659	6.682	6.376
**Binding χ^2**	0.884	1.163	0.971	1.062	0.909	1.924

Calculated first component lifetime τ1 and mean intensity waited lifetime τ_{avg} of fluorescein mixed with 100 µg/ml GO or PGNS after the addition of 0, 0.5, and 1 mM phenformin. Δ Is the change in τ from control with no phenformin added. The goodness of fit is calculated using Chi-squared (X2) for the lifetime decay

Table S3 *FLIM-FRET fit values for fluoresceine decays with GO and PGNS (as above).*

Sample	phenformin (mM)	Donor Lifetime ns	FLIM FRET Efficiency %	Apparent FRET Efficiency %	Donor-Acceptor Distance nm	Binding %	χ^2
**GO**	0	2.990	25.257	3.490	1.198	17.659	1.062
**GO**	0.5	1.761	55.967	1.711	0.961	6.682	0.909
**GO**	1	0.901	77.476	1.171	0.814	6.376	1.924
**PGNS**	0	3.143	21.418	5.813	1.242	32.151	0.884
**PGNS**	0.5	2.992	25.209	6.208	1.199	30.396	1.163

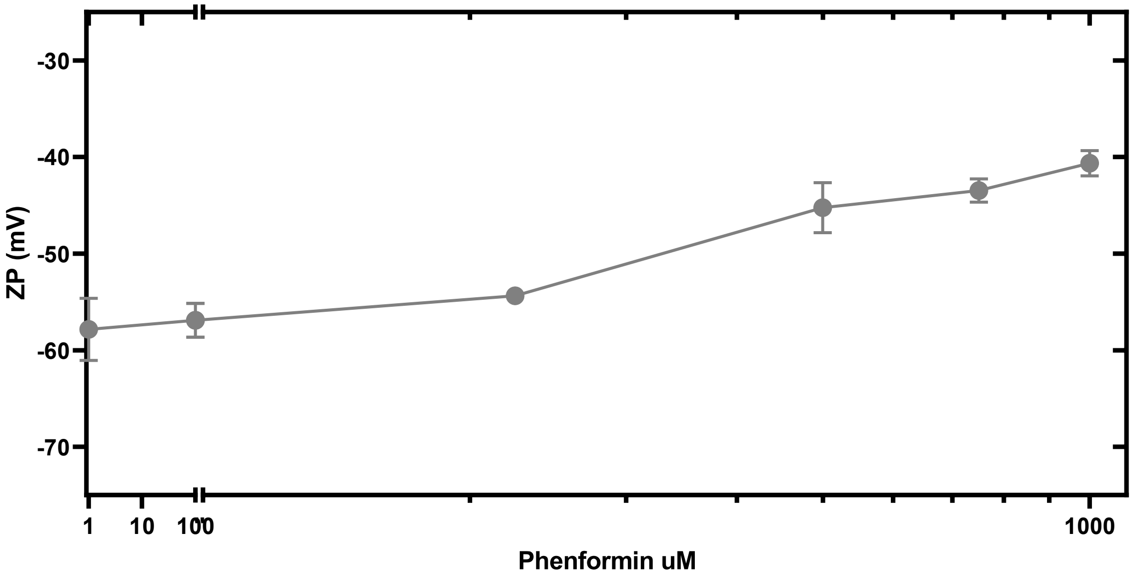

Figure S6 *Zeta potential changes towards more positive charges of carboxymethylcellulose (CMC), a model polymer rich in COOH, indicating its interaction with phenformin.*

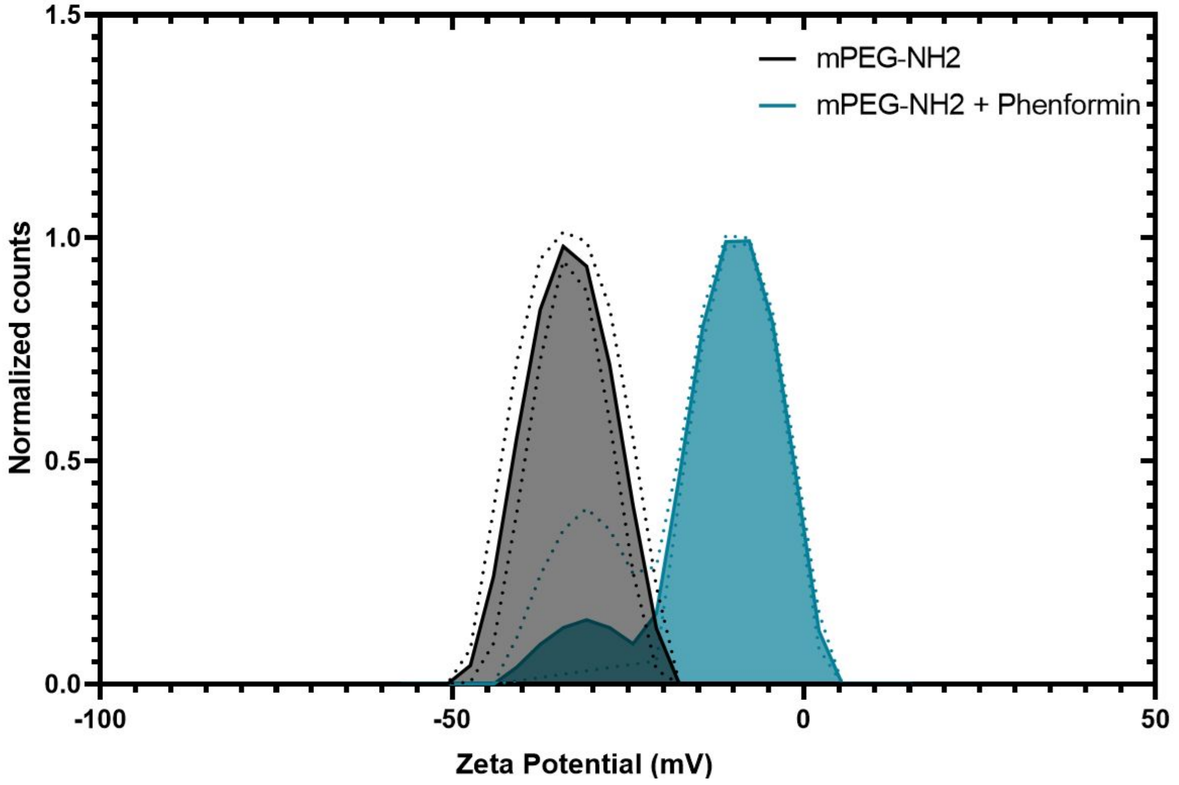

Figure S7 *Zeta-potential of mPEG-amine in water before and after adding phenformin. The neutralization of the PEG charge is an indication of its interaction with phenformin.*
